# Editorial: The heterogeneity of psychiatric symptoms and disorders

**DOI:** 10.3389/fpsyt.2026.1819690

**Published:** 2026-06-05

**Authors:** Yicheng Long

**Affiliations:** Department of Psychiatry, and National Clinical Research Center for Mental Disorders, The Second Xiangya Hospital, Central South University, Changsha, Hunan, China

**Keywords:** clinical symptom, heterogeneity, neuroimaging, psychiatric disorder, transdiagnostic

## Introduction

The clinical and biological heterogeneity in many common psychiatric disorders has garnered increasing research interest in recent years. It is increasingly recognized that different causal mechanisms may relate to the same disorder, and multiple outcomes of interest can occur within one individual. For instance, several clinical symptom-based subtypes have been identified using data-driven methods in major depressive disorder (MDD), one of the most common disorders worldwide (1). Genetic and neuroimaging studies have also provided ample evidence that MDD is biologically heterogeneous, involving a wide range of genes and brain circuits (2, 3). Additionally, studies have identified sex and age as notable contributing factors to the heterogeneity of MDD (4–6). Beyond MDD, significant research efforts have also focused on the heterogeneity of other conditions, such as schizophrenia (7), autism spectrum disorder (8), and bipolar disorder (9), as well as subclinical depressive/psychotic symptoms (10, 11). This collective effort underscores the critical importance of investigating the heterogeneity of psychiatric symptoms and disorders to clarify underlying pathophysiological mechanisms and improve treatment outcomes.

The goal of this Research Topic was to bring together multidisciplinary evidence that further dissects the heterogeneity across common psychiatric symptoms and disorders. We sought to move beyond simply acknowledging its existence to actively exploring its sources and implications. The response to this call has been very positive, and we are proud to present a collection of 21 articles that significantly advance this mission. These articles include original research, comprehensive reviews, commentary, and insightful case reports. In what follows, we synthesize the contributions along three interconnected dimensions: (1) clinical and phenomenological heterogeneity (symptom profiles, comorbidity patterns, and individual-level presentations); (2) biological and mechanistic heterogeneity (neuroimaging, omics, and functionally grounded frameworks); and (3) transdiagnostic and continuum-based heterogeneity (subclinical phenomena, cross-diagnostic risk factors, and early intervention).

## Clinical and phenomenological heterogeneity: from symptoms to comorbidity to the individual

Several contributions in this Research Topic examine heterogeneity through the lens of symptom presentation and comorbidity patterns. Using a data-driven cluster analysis in a large sample of psychosis patients (n=242), Lewandowski et al. identified four distinct motivational profiles (e.g., “High Avoidance”, “High Approach”) that cut across traditional diagnoses of schizophrenia spectrum and mood disorders with psychosis. Notably, these profiles differed significantly in depression, anxiety, and hedonic capacity, but not in community functioning – a finding that challenges the assumption that motivational subtypes automatically translate into real-world outcomes. In a different clinical context, Uygur demonstrated that insomnia severity in adults with attention deficit hyperactivity disorder (ADHD) is predicted by sleep reactivity, ADHD symptom severity, and female sex. This suggests that even within a single diagnosis, the drivers of a common comorbid symptom (insomnia) are heterogeneous and potentially sex-specific.

Comorbidity patterns themselves were a major focus. Hua et al. surveyed 707 patients undergoing gastroscopy in a general hospital and found that while 51 met criteria for major depression and 34 for depression-anxiety comorbidity, gastroenterologists detected only 3.92% of depression cases and zero cases of depression-anxiety. This striking detection gap highlights how clinical heterogeneity can be systematically overlooked in non-psychiatric settings. Bloch and Misiak used network analyses to map problematic online behaviors (POBs) in young adults and discovered three distinct communities – two of POBs and one of general psychopathology. Importantly, obsessive-compulsive symptoms emerged as the strongest predictor of other POBs, suggesting a potential target for early intervention.

The Research Topic also includes several case reports that illustrate heterogeneity at the individual level – often in extreme or treatment-resistant forms. Xie et al. reported a patient with schizophrenia who took super-dose clozapine (18 to 107 tablets/day, 25 mg/tablet) for five months due to irregular follow-up, highlighting the dangers of unsupervised medication escalation. Lai et al.described a patient with obsessive-compulsive disorder (OCD) with obsessional slowness who received anterior capsulotomy and subsequent accumbensotomy, only to have symptoms fully rebound by 30 months – a cautionary tale that certain heterogeneous subtypes of OCD may not benefit from neurosurgery. On the positive side, Connors et al. provided longitudinal follow-up of a patient with Down syndrome regression disorder and catatonia, showing that while intravenous immunoglobulin yielded only a small unsustained response, electroconvulsive therapy produced a rapid and sustained improvement. Together, these case reports demonstrate that individual-level heterogeneity is not merely anecdotal noise but carries critical lessons for treatment selection and safety.

## Biological and mechanistic heterogeneity: from brain structure to omics

Several articles directly investigated biological substrates of heterogeneity. Zhou et al. compared gray matter volume (GMV) among bipolar depression (BD), unipolar depression (UD), and healthy controls. While both patient groups showed reduced GMV in right superior temporal gyrus and right insula, the right posterior cingulate cortex (PCC) showed decreased GMV in BD but increased GMV in UD. This opposite direction of PCC abnormality – a potentially replicable biomarker – illustrates that what appears similar at the symptomatic level (depression) can have divergent neural signatures. Schroeder et al. examined borderline personality disorder (BPD) and found that dissociative experiences fully mediated the relationship between childhood trauma and auditory verbal hallucinations, but not delusional thoughts. This specificity suggests that within BPD, different psychotic symptoms arise through distinct pathways, reinforcing the need to parse heterogeneity at the mechanism level.

At a broader conceptual level, Stolfi et al. provided a comprehensive review of multi-omics approaches (genomics, epigenomics, transcriptomics, proteomics, metabolomics, gut microbiomics, immunomics) in MDD, arguing that integrating these data into a “patient fingerprint” using artificial intelligence could transform the field from a one-size-fits-all approach to true precision medicine. Rohlfing et al. proposed a “Psychotherapy Research Domain Criteria (RDoC)” framework, arguing that biologically grounded functional mechanisms – particularly needs and reward processing – can serve as a bridge between nomothetic science and idiographic treatment. These conceptual papers converge on a shared message: biological heterogeneity is not a nuisance to be controlled for, but the very target of investigation.

## Transdiagnostic and continuum-based heterogeneity: beyond diagnostic boundaries

A third major theme emerging from this Research Topic is that heterogeneity exists on a continuum, not solely within diagnosed clinical populations. Kim et al. conducted a two-wave longitudinal study during the pandemic and found that intrusive rumination acted as a transdiagnostic mediator of both internalizing and externalizing symptoms, with the exception of mania. This finding positions event-related rumination as a potential intervention target that cuts across diagnostic boundaries. Shin and Park used network analysis to examine depression-anxiety comorbidity in a community sample of 600 middle-aged adults. They found that perceived stress and mastery were the most central bridge factors linking physical health, cognitive adaptation, and social connection to comorbid symptoms. Notably, the connection between physical health and anhedonia was stronger in men, while social connection with family was linked to interpersonal problems only in women – underscoring gender-specific pathways.

Two systematic reviews directly addressed transdiagnostic risk. Lo Buglio et al. mapped 33 studies assessing both clinical high risk for psychosis (CHR-P) and borderline personality disorder (BPD), identifying four distinct research paradigms and offering ten concrete recommendations for future research, including the need for transdiagnostic interventions and cost-effectiveness evaluations. Argyriou et al. conducted a scoping review on the Hierarchical Taxonomy of Psychopathology (HiTOP) as a framework for personalized treatment selection in internalizing disorders, finding preliminary evidence but also major gaps such as inconsistent measurement and risk of Type I/II error. Together, these reviews argue that moving beyond categorical diagnosis toward dimensional, transdiagnostic models is not only theoretically appealing but empirically necessary.

## What this Research Topic collectively advances

Taken together, the 21 papers published in this Research Topic weave a compelling narrative. They confirm that investigating heterogeneity is not an academic exercise but an essential step toward clarifying mechanisms and improving treatment. More specifically, we believe the single most important shift enabled by this Research Topic is the recognition that heterogeneity is not noise but signal – and that parsing this signal requires moving from categorical diagnoses to multi-level, dimensional models that integrate clinical, biological, and transdiagnostic perspectives. The Research Topic also reveals important tensions: for example, motivational profiles did not predict community functioning (Lewandowski et al.), and neurosurgery for OCD with obsessional slowness led to full relapse (Lai et al.) – reminding us that biomarker or subtype discovery does not automatically translate into better outcomes. These tensions highlight where the field still falls short.

## Future directions

While this Research Topic makes substantial progress, several unanswered questions and practical next steps deserve emphasis. First, longitudinal designs are urgently needed to distinguish state versus trait heterogeneity – most studies here were cross-sectional. Second, multi-modal integration (e.g., combining neuroimaging, omics, and behavioral data in the same individuals) remains rare but will be necessary for clinically actionable stratification. Third, reproducibility across datasets and populations must become standard practice before subtypes can be adopted clinically. Fourth, stronger links between heterogeneity findings and treatment decision-making are required – for instance, can the four motivational profiles in psychosis (Lewandowski et al.) predict differential response to behavioral activation or medication? Finally, implementation science should address the detection gap documented by Hua et al., where gastroenterologists missed over 95% of depression cases. By embracing these challenges, we can move toward a future where psychiatric diagnosis and care are as unique as the individuals we serve.
